# Quantification of virus-infected cells using RNA FISH-Flow

**DOI:** 10.1016/j.xpro.2023.102291

**Published:** 2023-05-18

**Authors:** Cody J. Warren, Arturo Barbachano-Guerrero, Devra Huey, Qing Yang, Emma R. Worden-Sapper, Jens H. Kuhn, Sara L. Sawyer

**Affiliations:** 1 Department of Veterinary Biosciences, The Ohio State University, Columbus, OH 43210, USA; 2 BioFrontiers Institute, Department of Molecular, Cellular, and Developmental Biology, University of Colorado, Boulder, CO 80303, USA; 3 Integrated Research Facility at Fort Detrick, National Institute of Allergy and Infectious Diseases, National Institutes of Health, Fort Detrick, Frederick, MD 21702, USA

**Keywords:** Microbiology, Molecular Biology, In Situ Hybridization

## Abstract

We present a protocol to detect cells that have been infected by RNA viruses. The method, RNA fluorescence *in situ* hybridization flow cytometry (RNA FISH-Flow), uses 48 fluorescently labeled DNA probes that hybridize in tandem to viral RNA. RNA FISH-Flow probes can be synthesized to match any RNA virus genome, in either sense or anti-sense, enabling detection of genomes or replication intermediates within cells. Flow cytometry enables high-throughput analysis of infection dynamics within a population at the single cell level.

For complete details on the use and execution of this protocol, please refer to Warren et al. (2022).^1^

## Highlights

• Step-by-step procedure for virus-specific RNA FISH-Flow probe generation
• Detailed procedure for RNA FISH-Flow probe hybridization to virus-exposed cells
• Analysis of viral RNA-positive cells by flow cytometry

## Abstract (editor highlights)

Publisher’s note: Undertaking any experimental protocol requires adherence to local institutional guidelines for laboratory safety and ethics.

## Graphical abstract

**Figure:**
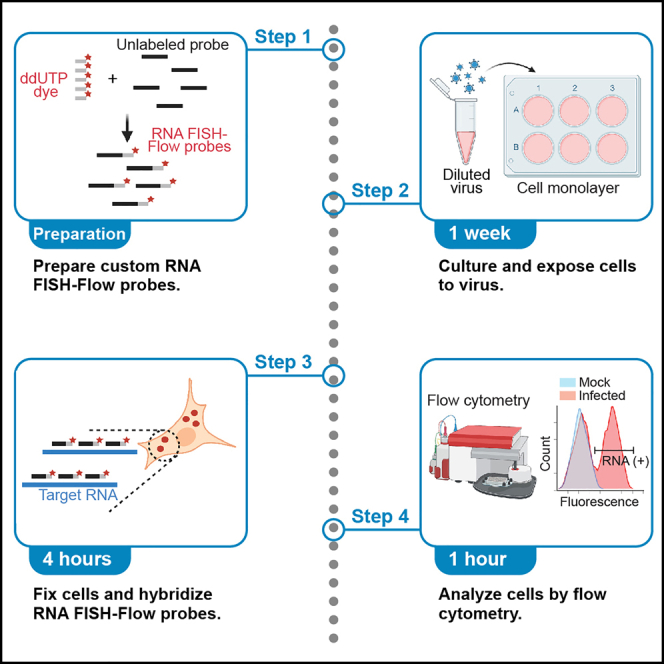

## Before you begin

**Figure 1:**
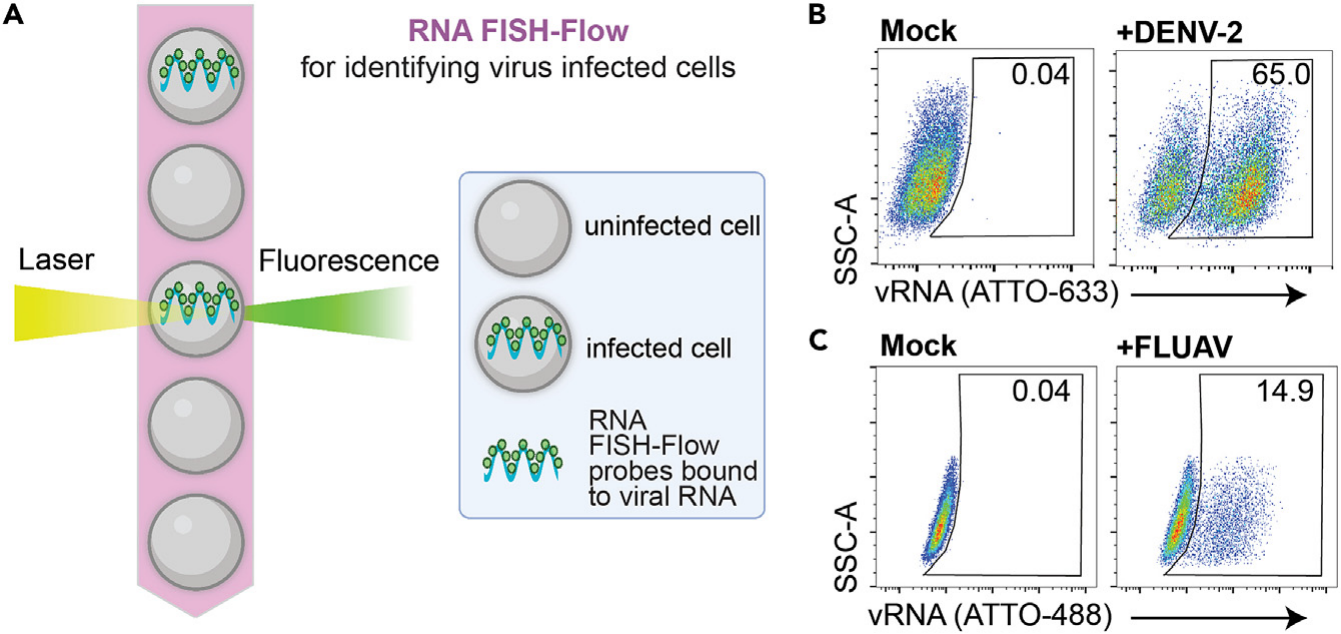
RNA FISH-Flow enables highly sensitive detection of virus infected cells by flow cytometry (A) Cartoon schematic of cells passing in single file through a flow cytometer machine. When fluorescently labeled RNA FISH-Flow probes, bound to viral RNA (vRNA), are excited by a laser, they emit a fluorescent signal that is then recorded by highly sensitive detectors. (B) A549 cells were infected with dengue virus 2 (DENV-2; strain 16681^2^) at a multiplicity of infection (MOI) of 0.5. (C) MDCK cells were infected with influenza A virus (FLUAV; A/H3N2/Udorn/72^3^) at an MOI of 0.1. Samples in (B) were hybridized with a DENV-2 NS5-specific ATTO-633 probe set and (C) were hybridized with a FLUAV segment 2-specific ATTO-488 probe set. All flow-cytometric events were first gated on forward vs side scatter (FSC-A × SSC-A) properties, followed by singlet discrimination. All gates were drawn based on mock-treated cells, and the percentage of cells positive for vRNA were derived.

This protocol describes the specific steps for detecting virus-infected cells using RNA fluorescence *in situ* hybridization flow cytometry (RNA FISH-Flow). This process uses 48 short (18–22-nucleotide-long), tandemly arrayed fluorescently labeled DNA probes that hybridize to viral RNA (vRNA) molecules. The cumulative signal of these probes hybridizing to a single strand of vRNA enables highly sensitive vRNA detection in cells. Cells are analyzed by flow cytometry, which enables high-throughput, single-cell-level quantification of virus-infected cells (Figure 1A).

We provide specific steps for performing RNA FISH-Flow on cells exposed to simian hemorrhagic fever virus (SHFV) as described in Warren et al.^1^ SHFV is an understudied virus of potential zoonotic concern; consequently, few serological reagents are available for its molecular characterization in the laboratory. Thus, RNA FISH-Flow served as a valuable tool for quantifying cells infected by SHFV. This protocol can also be readily adapted to study infection by any single-stranded RNA virus. Simply modify the cell type, culture conditions, and RNA FISH-Flow probe set as necessary. To exemplify this point, cells were exposed to dengue virus 2 (DENV-2) or influenza A virus (FLUAV) and then the cells testing positive for vRNA were enumerated 24 h later by RNA FISH-Flow. The vRNA signal is readily detected in cells exposed to virus (Figures 1B and 1C). This highlights the adaptability of this technique and potential breadth of viral infections that can be studied using RNA FISH-Flow.

### Institutional permissions

The use of SHFV, DENV-2, and FLUAV in this study was approved by biosafety committees at the University of Colorado Boulder and The Ohio State University. Appropriate biosafety trainings were completed before starting experiments.

### Probe design

**Timing: 5 min**

1. Design probes.
  a. Create an account at the LGC Biosearch Technologies website: https://www.biosearchtech.com/stellaris-designer.
  b. Log in with username and password.
  c. Complete the required fields.
    i. For organism, select “Other.”
    ii. Masking level defaults to “2.”
    iii. Set number of probes to “48,” oligo length to “20,” and min spacing length to “2.”
  d. Enter the target sequence into the provided field. The target sequence should be 1–4 kb in length.
  e. Select “Design probes.”

**Figure 2:**
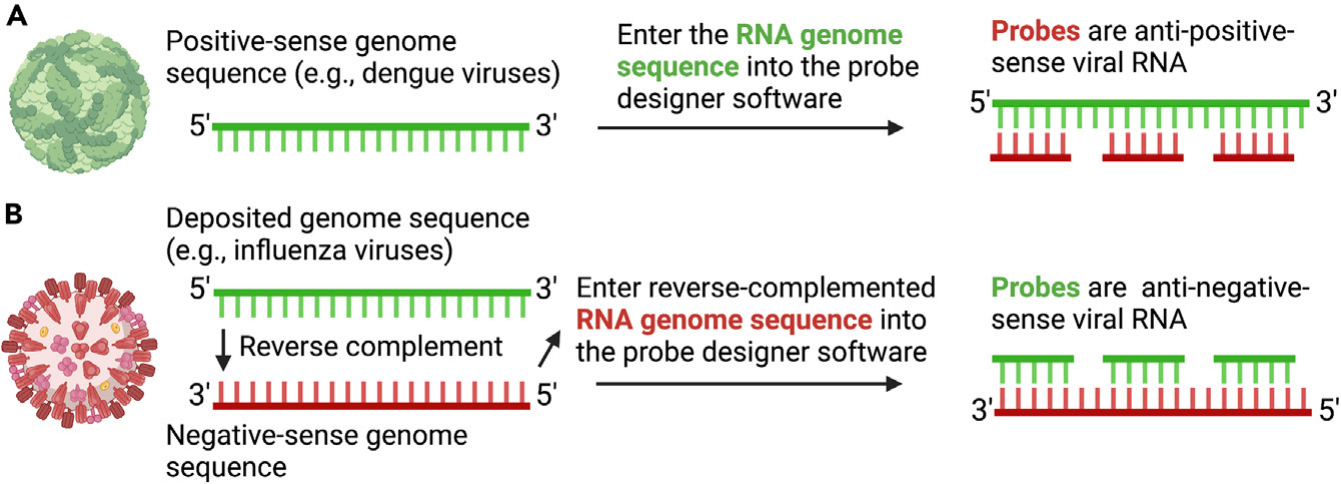
Strategy for RNA FISH-Flow probe design By convention, for RNA viruses, the sequences deposited in databases (e.g., GenBank) are entered as the coding (positive-sense) strand, using Ts instead of Us. To design RNA FISH-Flow probes to a specific RNA virus genome, the probes must have a complementary sequence to the actual target RNA molecule. The design tool replaces any Us with Ts if they are in the input sequence. (A) Database sequences that are deposited for positive-sense RNA viruses (e.g., dengue viruses) are already in coding strand, and thus RNA FISH-Flow probes designed anti-sense to the target sequence will hybridize. (B) However, for detection of negative-sense viruses (e.g., influenza viruses), the database sequence must first be reverse-complemented. Only then will the designed RNA FISH-Flow probes hybridize to the negative-sense genome target.

**CRITICAL:** The tool designs probes that are antisense to the input sequence. If the aim is to detect positive-sense vRNA, enter the 5'→3′ DNA target sequence as is; the probes will be anti-positive-sense vRNA (Figure 2A). If the aim is to detect negative-sense replication intermediates/antigenomes (or a negative-sense virus), enter the reverse complement of the sequence so the design tool generates probes in the correct orientation (Figure 2B).

2. From the “Review design results” screen, copy and paste the probe sequences into a spreadsheet.
3. Arrange the oligos in a 96-well plate format from the appropriate vendor (for instance, IDT). When ordering, have the vendor add water to a final concentration of 200 μM to simplify pipetting.

***Note:*** We find fluorophore conjugation to DNA oligonucleotides to be a simple, inexpensive, and more scalable alternative to ordering pre-conjugated probes. More information on how to directly conjugate fluorophores to these probes is described below.

***Alternatives:*** After selecting “Design probes,” the “Review design results” screen displays. By selecting “Order” the probes can be purchased directly through LGC Biosearch Technologies. Select a desired fluorophore and add the item to the cart.

### Probe labeling

**Timing: 1 d**

Pre-conjugated fluorophore probes are costly and, further, the fluorophores offered for probe conjugation by commercial vendors are limited. Here, we describe a simple method for direct fluorophore conjugation to DNA oligonucleotides.

4. Set up the labeling reaction.
  a. Order DNA oligonucleotides per the guidelines described in “probe design,” above.
  b. Pool 10 μL of each oligo in a single tube and mix by pipetting.
    ***Note:*** This oligo mix can be stored at −20°C and used for future labeling reactions.
  c. Set up the labeling reaction mixture:
    **Table:** Probe labeling reaction mixture

Reagent	Final concentration	Amount
5× Reaction buffer	1×	10 μL
Terminal deoxynucleotidyl transferase (TdT) (20 U/μL)	20 U	1 μL
Fluorescent dye-labeled 5-propargylamino-2′,3′-dideoxyuridine-5′-triphosphate (ddUTP) (1 mM)	60 μM	3 μL
Oligonucleotide mix (200 μM)	20 μM	5 μL
UltraPure Molecular Biology Grade Water DEPC-Treated	N/A	31 μL
**Total**	**N/A**	**50 μL**

    ***Note:*** Choose the 5-propargylamino-20,30-dideoxyuridine-50-triphosphate (ddUTP) that best suits the specific application and instrumentation. There are many fluorescent dye-labeled ddUTPs available commercially from Jena Bioscience (https://www.jenabioscience.com/nucleotides-nucleosides/nucleotides-by-structure/fluorescent-nucleotides/uridines-dye-labeled/5-propargylamino-ddutp). We use ddUTPs labeled with ATTO dyes (ATTO-TEC) due to their high absorption, high fluorescence emission, and high photostability. We have used ATTO-488, ATTO-550, and ATTO-633 with success.
  d. Incubate the labeling reaction mixture at 37°C for 8 h in a PCR machine with the hot-lid set to 37°C.
  e. Add 0.75 μL of additional 20 U/μL terminal deoxynucleotidyl transferase (TdT), mix gently, and continue the incubation for 24 h total.

5. Purify the fluorescent dye-labeled DNA probes.
  a. Pre-chill 100% ethanol on ice for at least 1 h.
  b. To the probe labeling reaction add 50 μL of 3 M sodium acetate (CH₃COONa; NaOAc).
  c. Add 400 μL of ice-cold 100% ethanol and mix well by pipetting.
  d. Store the mixture at −80°C for 30 min.
  e. Centrifuge the microfuge tube at >18,000 ×*g* for 15 min at 4°C.
    ***Note:*** A small pellet should be visible at the bottom of the microfuge tube. Take care to avoid dislodging this pellet during subsequent wash steps.
  f. Carefully remove the liquid using a pipette and replace with 500 μL of ice-cold 70% ethanol.
  g. Centrifuge the microfuge tube at >18,000 ×*g* for 1 min at 4°C.
  h. Repeat wash steps 5f–g.
  i. Carefully remove the liquid using a pipette.
  j. Air dry the pellet by inverting the microfuge tube (lid side down with lid open), and place it on a paper towel protected from light. Allow at least 5 min for the ethanol to evaporate.
  k. Resuspend the pellet with 50 μL of UltraPure Molecular Biology Grade Water DEPC-Treated.
  l. Aliquot 5 μL of the labeled probes into PCR strip tubes and store at −20°C.
    ***Note:*** Throughout this protocol, UltraPure Molecular Biology Grade Water DEPC-Treated was used when generating mixtures. It is important that the water is free of nucleases to avoid degradation of the nucleic acid probes. Alternatively, nuclease-free water (not DEPC-treated) may be used as a substitute (e.g., Invitrogen Cat# AM9932).
    ***Note:*** Fluorescent dye-labeled probes are light-sensitive. Avoid exposure to bright light. Store samples in light-protected boxes.

### Quality control: spectroscopic analysis of RNA FISH-Flow probes

**Timing: 10 min**

In the probe labeling procedure, ddUTPs labeled with ATTO dyes (ATTO-TEC) were used. These dyes are conjugated to the 3′ end of DNA oligonucleotides in a reaction catalyzed by the TdT enzyme. Several methods for measuring the efficiency of probe labeling have been described, including polyacrylamide gel electrophoresis (PAGE) densitometry and fluorescence spectroscopy.^4^^,^^5^ Here, we describe a simple spectroscopic method for determining whether the probes have been fluorescently labeled.

6. Generate samples of labeled and unlabeled probes.
  a. After purifying labeled RNA FISH-Flow probes, resuspend in 50 μL of UltraPure Molecular Biology Grade Water DEPC-Treated (see section on probe labeling, step 5k).
  b. Generate an unlabeled oligonucleotide mixture by adding 5 μL of unlabeled oligonucleotides (see section on probe labeling, step 4b) with 45 μL of UltraPure Molecular Biology Grade Water DEPC-Treated.
7. Measure the concentration of single-stranded DNA (ssDNA) in ng/μL for the labeled RNA FISH-Flow probes and unlabeled oligonucleotide mixture using a spectrophotometer (e.g., Nanodrop or similar instrument).
8. Calculate the percent recovery as follows: %recovery = 100 × (c_labeled_/c_unlabeled_) in which “c” is the ssDNA concentration in ng/μL.

**Figure 3:**
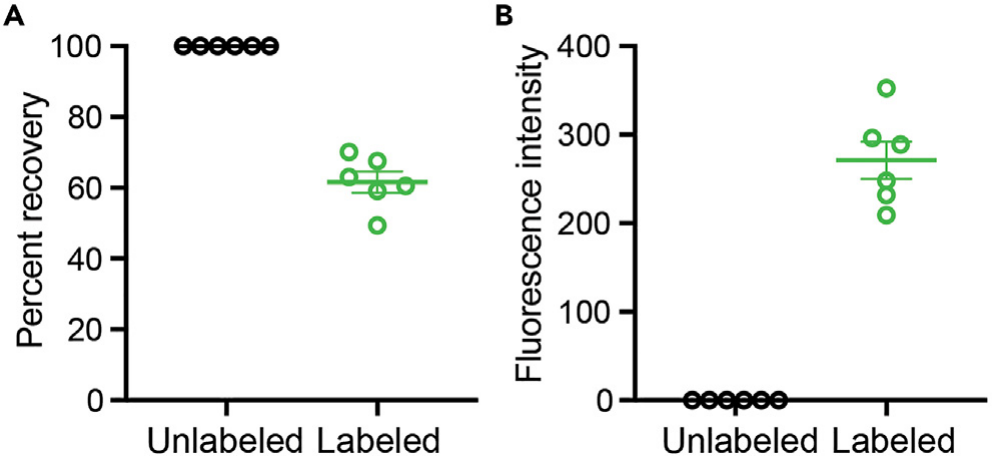
Recovery and labeling efficiency of purified RNA FISH-Flow probes Six independent oligonucleotide pools were designed and ATTO-488 labeled (or not) as described in the “probe design” and “probe labeling” sections. (A) Single-stranded DNA (ssDNA) concentrations of labeled and unlabeled RNA FISH-Flow probe mixtures were determined using a Nanodrop instrument. The percent recovery was calculated for each matched pair (n = 6). (B) Raw fluorescence values were acquired with a Synergy LX analyzer and the Take3 accessory. A ssDNA concentration corrected fluorescence intensity value was calculated for each labeled and unlabeled RNA FISH-Flow probe mixture. Consistently, RNA FISH-Flow probe labeling resulted in higher fluorescence intensity values compared to unlabeled probe mixtures. Data are shown as the mean ± SEM from six independent pairs of labeled and unlabeled RNA FISH-Flow probe mixtures.

***Note:*** Based on analysis of multiple RNA FISH-Flow probe sets, recovery of 50–70% is to be expected following labeling and purification (Figure 3A). If the calculated percent recovery is lower than 50%, consider repeating the labeling reaction and purification. Proceeding with an RNA FISH-Flow probe set with a reduced recovery may negatively impact the sensitivity of the RNA FISH-Flow assay (see section on troubleshooting).

9. Using a spectrofluorometer, select excitation and emission filters compatible with the fluorescent dye used in the RNA FISH-Flow probe labeling step (e.g., excitation 485 nm / emission 528 nm in the case of ATTO-488 dye).
10. Blank the spectrofluorometer using the probe diluent (e.g., UltraPure Molecular Biology Grade Water DEPC-Treated).
11. Measure the fluorescence values for the unlabeled oligonucleotide mixtures and labeled RNA FISH-Flow probes.
12. Calculate a ssDNA concentration corrected fluorescence intensity value for both unlabeled oligonucleotide mixtures and labeled RNA FISH-Flow probes as follows: Fluorescence intensity = (f_unlabeled_ /c_unlabeled_) and Fluorescence intensity = (f_labeled_/c_labeled_) in which “f” is the fluorescence intensity reading in arbitrary units (a.u.).

***Note:*** The fluorescence values obtained will vary depending on the instrument model and acquisition settings (e.g., gain and detector sensitivity). Use the same instrument and settings when comparing batches of labeled RNA FISH-Flow probes. The fluorescent ratio values for labeled RNA FISH-Flow probes should be higher than for the unlabeled oligonucleotide mixtures (Figure 3B).

## Key resources table

**Table:** 

REAGENT or RESOURCE	SOURCE	IDENTIFIER
**Bacterial and virus strains**		

Simian hemorrhagic fever virus (SHFV) isolate NIH LVR42-0/M6941	American Type Culture Collection (ATCC)	Cat# VR-533
Dengue virus 2 (DENV-2; 16681)	Kinney et al.^2^	N/A
Influenza A virus (FLUAV; A/H3N2/Udorn/72)	Chen et al.^3^	N/A

**Recombinant DNA**		

Wild-type recombinant SHFV (rSHFV)-encoding cDNA-launch plasmid	Cai et al.^6^	N/A
rSHFV expressing enhanced green fluorescent protein (rSHFV-eGFP)-encoding cDNA-launch plasmid	Cai et al.^6^	N/A

**Chemicals, peptides, and recombinant proteins**		

Eagle’s minimum essential medium (EMEM)	ATCC	Cat# 30-2003
10% fetal bovine serum (FBS)	MilliporeSigma	Cat# TMS-013-B
1× penicillin-streptomycin solution (Pen Strep)	Avantor	Cat# 45000-652
32% paraformaldehyde (PFA)	VWR	Cat# 100504-858
100% ice-cold ethanol (EtOH)	Thermo Fisher Scientific	Cat# 04-355-223
5-propargylamino-2′,3′-dideoxyuridine-5′-triphosphate (ddUTP) ATTO-633	Jena Bioscience	Cat# NU-1619-633
5-propargylamino-2′,3′-dideoxyuridine-5′-triphosphate (ddUTP) ATTO-550	Jena Bioscience	Cat# NU-1619-550
5-propargylamino-2′,3′-dideoxyuridine-5′-triphosphate (ddUTP) ATTO-488	Jena Bioscience	Cat# NU-1619-488
Phosphate-buffered saline (PBS)	Caisson Labs	Cat# PBL01-6X500ML
Terminal deoxynucleotidyl transferase (TdT) (20 U/μL)	Thermo Fisher Scientific	Cat# EP0161
5× Reaction buffer	Thermo Fisher Scientific	Cat# EP0161
Sodium acetate 3M (CH₃COONa; NaOAc)	Amresco	Cat# E498-200ML
Dextran sulfate 50% solution	MilliporeSigma	Cat# S4030
Deionized formamide	MilliporeSigma	Cat# S4117
20× nuclease-free saline-sodium citrate (SSC)	Thermo Fisher Scientific	Cat# AM9770
Trypsin ethylenediaminetetraacetic acid (EDTA)	Corning Incorporated	Cat# 25-052-Cl
0.5 M EDTA	MilliporeSigma	Cat# 324506-100ML
UltraPure Molecular Biology Grade Water DEPC-Treated	IBI Scientific	Cat# IB42201

**Experimental models: Cell lines**		

MA-104 Clone 1	ATCC	Cat# CRL-2378.1
MDCK	ATCC	Cat# CCL-34
A549	ATCC	Cat# CCL-185

**Software and algorithms**		

Stellaris probe designer	Biosearch Technologies	https://www.biosearchtech.com/stellaris-designer
FlowJo v10.8.0 software	BD Life Sciences	N/A
Reverse complement	N/A	https://www.bioinformatics.org/sms/rev_comp.html

**Other**		

96-well v-bottom plate	NEST Scientific	Cat# 701211

## Materials and equipment

### Wash buffer A

• Wash buffer A solution: Add 15 mL of 20× nuclease-free saline-sodium citrate (SSC) to 120 mL UltraPure Molecular Biology Grade Water DEPC-Treated (2.2× SSC final concentration). Filter using a 0.22-μm filter.

***Note:*** The buffer solution can be stored at 4°C for up to 1 yr.

• Deionized formamide: Add 1 volume of deionized formamide to 9 volumes of wash buffer A just prior to use (2× SSC and 10% formamide final concentration). Prepare fresh for each experiment.

***Note:*** Formamide should be stored according to manufacturer-recommended conditions to maintain a deionized state.

***Alternatives:*** We have also had success with Stellaris RNA FISH Wash Buffer A, purchased from Biosearch Technologies (Cat# SMF-WA1-60). Combine 2 mL wash buffer A, 7 mL of UltraPure Molecular Biology Grade Water DEPC-Treated, and 1 mL of deionized formamide just prior to use. Prepare fresh for each experiment; scale as needed.

**CRITICAL:** Deionized formamide is a carcinogen. Work in a fume hood to avoid breathing vapors. Avoid mixing techniques that generate aerosols (i.e., pipette instead of vortex). Avoid direct skin contact. Collect all waste containing deionized formamide and dispose according to institutional environmental health and safety guidelines.

### Wash buffer B

• Wash buffer B solution: Add 15 mL of 20× nuclease-free SSC to 135 mL UltraPure Molecular Biology Grade Water DEPC-Treated (2× SSC final concentration). Filter using a 0.22-μm filter.

***Note:*** The buffer solution can be stored at 4°C for up to 1 yr.

***Alternatives:*** We have also had success with Stellaris RNA FISH Wash Buffer B, purchased from Biosearch Technologies (Cat# SMF-WB1-20).

**Table:** Hybridization buffer

Reagent	Final concentration	Amount
Dextran sulfate 50% solution	10%	2 mL
Deionized formamide	10%	1 mL
20× nuclease-free saline-sodium citrate (SSC)	2×	1 mL
UltraPure Molecular Biology Grade Water DEPC-Treated	N/A	6 mL
**Total**	**N/A**	**10 mL**

***Note:*** Filter using a 0.22-μm filter. Aliquot and store at −20°C for up to 1 yr.

***Alternatives:*** We have also had success with Stellaris RNA FISH hybridization buffer, purchased from Biosearch Technologies (Cat# SMF-HB1-10).

### Fixation buffer

• Fixation buffer: Dilute paraformaldehyde (PFA) to a 4% final concentration in phosphate-buffered saline (PBS). Aliquot before storage.

***Note:*** Store at −20°C for 1 yr or 4°C for 1 week.

### Permeabilization solution

• Permeabilization solution: Dilute ethanol to 70% final concentration in deionized water (diH_2_O).

***Note:*** Store at ambient temperature and chill on ice for 1 h just prior to use.

### Media

• Media supplemented with fetal bovine serum (FBS) and antibiotics: Mix Eagle’s minimum essential medium (EMEM) with 10% FBS (for normal cell growth) or 2% FBS (during virus infections) and 1× penicillin-streptomycin solution.

***Note:*** Follow manufacturers’ shelf-life recommendations. Store at 4°C.

***Note:*** Use media appropriate for your cultured cell lines.

### Fluorescence-activated cell sorting (FACS) buffer

• FACS buffer: Dilute FBS (to a 2% final concentration) in PBS and add ethylenediaminetetraacetic acid (EDTA; 1 mM final concentration). Use sterile reagents or a filter to sterilize before storage.

***Note:*** Store at 4°C for up to 1 yr.

### Flow cytometry

• Several flow cytometers were used for data acquisition. These include the Accuri C6 Cytometer (BD) and the Attune NxT acoustic focusing cytometer (Life Technologies). At least 20,000 cell events were collected following singlet discrimination. Data were analyzed with FlowJo v10.8.0 (Becton, Dickinson and Company).

**CRITICAL:** When selecting fluorophores for oligo conjugation, it is important to understand the configuration of the flow cytometer. Knowledge of the number and types of lasers and filters will help determine which fluorophores are compatible for use.

### Spectroscopic analysis

• A Synergy LX multimode analyzer (spectrofluorometer; BioTek, Agilent) was used to calculate the ssDNA concentration (260 nm absorption) and the fluorescence (excitation 485 nm/ emission 528 nm filter cube) of the unlabeled oligonucleotide mixtures and labeled RNA FISH-Flow probes.
• A Take3 Microvolume Plate (BioTek, Agilent) was used with the Synergy LX analyze to minimize the sample volume needed for spectroscopic analysis.

## Step-by-step method details

### Seeding of cells in 6-well plates

**Timing: Approximately 30 min, depending on the number of plates seeded**

This major step explains the seeding of target cells in 6-well plates used for virus infections.

***Note:*** While these steps are specific to experiments involving simian hemorrhagic fever virus (SHFV) as outlined in Warren et al.,^1^ they are also generalizable. Time, media composition, and culturing conditions can be altered, depending on the cell line being used and the virus being tested.

1. Wash a confluent 10-cm dish (or T75 flask) of MA-104 Clone 1 cells with 10 mL of PBS and rock gently to wash the entire monolayer thoroughly.
2. Add 1.5 mL of 0.05% trypsin EDTA and place the container of cells back in the incubator for 3–5 min.
3. Neutralize the trypsin with 8.5 mL of EMEM containing 10% FBS.
4. Count viable cells using trypan blue and an automated cell-counter or manually using a hemocytometer.
5. Dilute to 1.5 × 10^5^ live cells per mL in growth media and transfer 2 mL to each well of a 6-well plate (3 × 10^5^ live cells plated per well).

***Note:*** The number of wells needed depends on the experiment being performed. For instance, if vRNA production at 24 h is to be assessed, two conditions would be plated: one mock-treated and one virus-exposed. If vRNA production over a time course is to be assessed, ensure that each time point (1, 12, 24, and 48 h) has a paired mock condition (8 total wells). Furthermore, the number of cells plated will vary depending on the cell line being used. The goal is to plate cells to become confluent the next day. We find that plating 1 × 10^5^ to 1 × 10^6^ cells is ideal for RNA FISH-Flow, as some cells may be lost during sample processing.

6. Incubate cells overnight at 37°C and 5% CO_2_.

### Exposure of cells to virus

**Timing: Approximately 2 h**

This major step describes infection of target cells with simian hemorrhagic fever virus (SHFV). These conditions can be adjusted as necessary to accommodate testing of any RNA virus of interest.

7. Remove SHFV from −80°C storage and thaw at ambient temperature.
8. In a sterile microfuge tube, dilute virions into 300 μL of serum-free EMEM to achieve the desired multiplicity of infection (MOI). If exposing multiple wells of a 6-well plate, scale this volume up, as needed.

***Note:*** We use an MOI of 3 and 0.03 for single-step and multi-step growth curves, respectively. If assessing early events prior to vRNA replication is of interest (i.e., invading virions), an MOI >10 should be used.

9. Discard all growth media from the 6-well plate containing the monolayer of MA-104 cells.
10. Wash the plate one time with PBS.
11. Remove the PBS wash and add 300 μL of media with diluted virions (or media alone for mock treatment).
12. Rock the plate gently and return it to the incubator.
13. Continue to rock the plate gently every 15 min for 1 h to ensure that the cells do not dry up.
14. Aspirate the virion inoculum (or media alone for mock) from each well.

**CRITICAL:** To minimize plastic waste, remove the virion inoculum without changing tips between wells. If multiple MOIs were tested, move from the most dilute sample (low MOI inoculum) to the least dilute sample (high MOI inoculum). Additionally, remove media from the mock-treated wells first to ensure that no carry-over contamination occurs from the virus-exposed to mock-treated samples.

15. Wash each well with 2 mL of PBS and rock gently to thoroughly wash the entire monolayer.
16. Discard the PBS wash.
17. Wash and discard two more times.
18. Replace the final PBS wash with 2 mL of EMEM + 2% FBS and return the plate to the incubator.
19. Incubate the cells at 37°C and 5% CO_2_ for the appropriate time (according to the experimental design).

### Fixation of cells following infection

**Timing: Approximately 2 h**

This major step describes fixation of cells following virus exposure. The time post-exposure when the cells are processed is dependent on the question being asked. For SHFV, exposure is stopped after 1 h to assess incoming vRNA genomes. This typically requires a high MOI (>10) and has limited resolution capacity because only a few RNAs will be detected per cell. As the vRNA is replicated within cells, fluorescence detection is more easily discriminated from background fluorescence. A time course of 4–12 h enables robust detection of virus-infected cells. Longer time courses (24–72 h) can also be used to measure spread through the cell-culture monolayer.

20. Remove the cell-culture media and wash the monolayer one time with PBS.
21. Add 0.5 mL of 0.05% trypsin EDTA and return the plate to the incubator.
22. Incubate the cells at 37°C and 5% CO_2_ for 3–5 min.
23. Neutralize the trypsin with 0.5 mL of EMEM containing 10% FBS.
24. Transfer the cells to a sterile 1.5-mL microfuge tube.
25. Pellet the cells by centrifugation for 5 min at 500 ×*g.*
26. Carefully remove the cell supernatant using a pipette, being sure to not dislodge the cell pellet.
27. Resuspend the cell pellet in 200 μL of PBS.
28. Transfer the cell suspension to 96-well v-bottom plates.

***Alternatives:*** 96-well v-bottom plates are used to enhance processivity when multiple samples are being handled simultaneously. This enables use of a multichannel pipette for the addition and removal of liquids from the samples. Alternatively, the protocol can be performed using microfuge tubes, especially when a small number of samples is being processed.

29. Pellet the cells by centrifugation for 5 min at 500 ×*g.*
30. Discard the PBS wash.

***Note:*** Multiple incubation and washing steps are used throughout this protocol. At any point during these steps, if care is not taken, it is possible that cell pellets could accidentally be discarded. If processing samples in a 96-well plate, we suggest that, following each centrifugation, the plate is tilted and liquid is removed from the sides of the wells using a multichannel pipette—taking care not to disturb the pellet. If using microcentrifuge tubes, carefully draw liquid out from the side of the tube opposite the pellet (avoid wide-mouth pipette tips).

31. Using a multichannel pipette, add 200 μL of fixation solution (4% PFA in PBS) to each well.
32. Mix by gently pipetting.
33. Incubate the cells in fixation solution for 10 min at ambient temperature.
34. Pellet the cells by centrifugation for 5 min at 500 ×*g.*
35. Remove the fixation solution and repeat the wash steps three times with 200 μL of PBS.
36. Remove the final wash and add 200 μL of ice-cold permeabilization solution (70% ethanol diluted in diH_2_O) to each well.
37. Mix by gently pipetting.
38. Store the plate at 4°C for at least 1 h.

**Pause point:** Cells can be stored in permeabilization solution at 4°C for up to 7 d, which is especially beneficial when processing samples at multiple time points. For instance, harvesting of different wells may be done at 4, 12, and 24 h post-exposure to visualize the kinetics of virus spread. Once the time course is complete, all permeabilized cells can be processed in one batch.

***Note:*** Extended permeabilization times may cause minor changes to the cells that affect their forward- and side-scatter properties. This may negatively impact downstream gating schemes used in flow cytometry; i.e., using one mock-treated well to generate gates may not be accurate if applied to several time points. To overcome this issue, harvest a mock-treated well alongside virus-exposed wells at each time point. In this way, each time point has a matched control that can be used for creating gates.

### Probe hybridization

**Timing: 2–24 h**

This major step describes the process of probe hybridization.

39. Pellet the cells by centrifugation for 5 min at 500 ×*g.*
40. Remove the permeabilization solution using a pipette and gently resuspend the cells in 200 μL of wash buffer A.
41. Pellet the cells by centrifugation for 5 min at 500 ×*g.*
42. While the cells are being centrifuged, prepare the hybridization buffer. A total of 49.5 μL of hybridization buffer and 0.5 μL of labeled probe is needed for each well. Make a master mixture that covers all samples, including the mock-treated wells.

***Note:*** We have had similar success further diluting the probe stock 1:10 in PBS and using this diluted mixture in a hybridization reaction (0.05 μL of probe stock per well). We recommend initial testing of different probe dilutions to determine the optimum quantity needed to (1) maintain sensitivity and (2) limit background staining.

43. Remove the wash buffer A and resuspend the cells in 50 μL of hybridization buffer containing the probe.

***Note:*** The hybridization buffer is very viscous, so take care to ensure that the cell pellet is gently but thoroughly resuspended.

44. Wrap the plate with parafilm and incubate protected from light at 37°C for at least 1 h.

**Pause point:** We have observed no difference in hybridizing cells for 1–4 h or overnight (≈16 h). Proceed with a hybridization time that is convenient for the experiment/schedule.

45. Remove the plate from incubator and add 150 μL of wash buffer A to each well.
46. Mix by pipetting.
47. Pellet the cells by centrifugation for 5 min at 650 ×*g.*
48. Using a multichannel pipette, carefully remove the wash solution, being sure to not dislodge the cell pellet.

***Note:*** From this point forward, the pellet may be easily dislodged from the plate and thus easily lost. Be very gentle with the pelleted cells. We suggest tilting the plate to draw liquid out from the sides of the wells and not near the pelleted cells, which are concentrated in the “v” of the plate wells.

49. Resuspend the cells in 200 μL of wash buffer A and incubate protected from light at 37°C for 30 min.
50. Pellet the cells by centrifugation for 5 min at 650 ×*g.*
51. Remove wash buffer A and resuspend the cells in 200 μL of wash buffer B.
52. Pellet the cells by centrifugation for 5 min at 650 ×*g.*
53. Remove wash buffer B and resuspend cells in 150 μL FACS buffer.
54. Proceed to flow-cytometry acquisition.

## Expected outcomes

When performed properly, this protocol is designed to enumerate cells positive for vRNA by flow cytometry. This technique can be applied to the study of viruses for which experimental resources are limited. Further, this technique avoids the reliance on commercially available kits, or viral clones that are modified to encode fluorescent reporter genes, thus saving time and effort.

**Figure 4:**
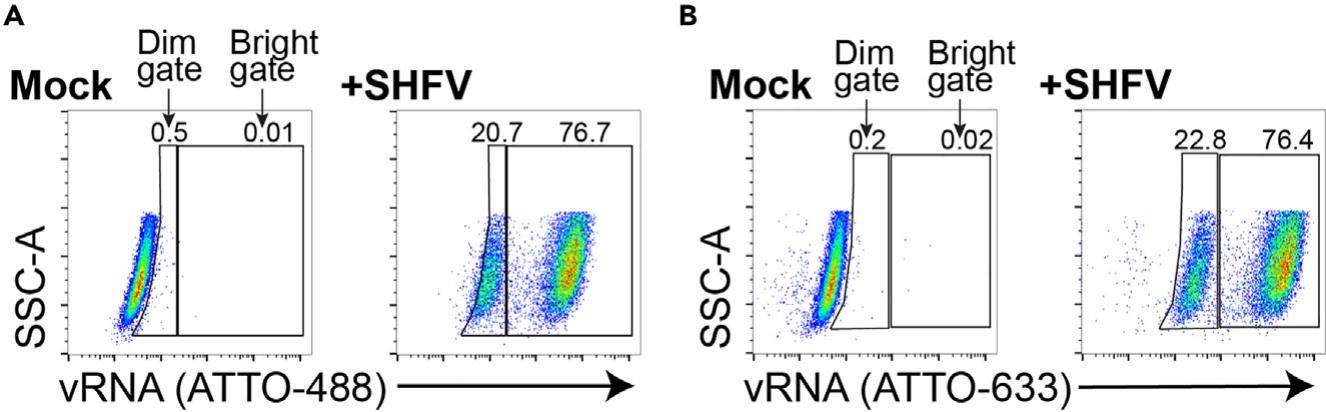
Example of results at 24 h post-exposure to virus MA-104 cells were exposed to simian hemorrhagic fever virus (SHFV) at a multiplicity of infection (MOI) of 3. At 24 h post-exposure, the cells were harvested and analyzed with RNA fluorescence *in situ* hybridization flow cytometry (RNA FISH-Flow). (A and B) Samples in (A) were hybridized with an SHFV ORF1a-specific ATTO-488 probe set and those in (B) were hybridized with the same probe set that was alternatively labeled with ATTO-633. All flow-cytometric events were first gated on forward vs side scatter (FSC-A × SSC-A) properties, followed by singlet discrimination. All gates were drawn based on mock-treated cells, and the percentage of cells positive for viral RNA (vRNA) in the dim and bright gates were derived.

Figure 4 shows data from an experiment in which MA-104 cells were exposed to SHFV and the cells testing positive for vRNA were enumerated 24 h later by RNA FISH-Flow. The same RNA FISH-Flow probe set was labeled with ATTO-488 (Figure 4A) or ATTO-633 (Figure 4B) using the methods described in this protocol. This figure illustrates several key points: First, a vRNA signal is readily detected in cells exposed to virus with very minimal background staining (comparing mock-exposed to virus-exposed panels). Second, independent probe labeling with two different fluorophores yielded near-identical results. Finally, we observed two unique patterns of fluorescence intensity in virus-exposed cells—dim and bright vRNA signals. Thus, we drew two gates based on the mock-treated cells: a “dim gate” that spanned ≈1 decade of fluorescence and a “bright gate” that spanned several decades of fluorescence. Given that this was a 24-h infection time course, we suspect that the dim signal represented cells newly infected by viruses (low vRNA levels), whereas the bright signal represented cells where virus had begun replicating (high vRNA levels). Alternatively, the dim signal could represent a small subpopulation of cells that poorly replicate vRNA.

**Figure 5:**
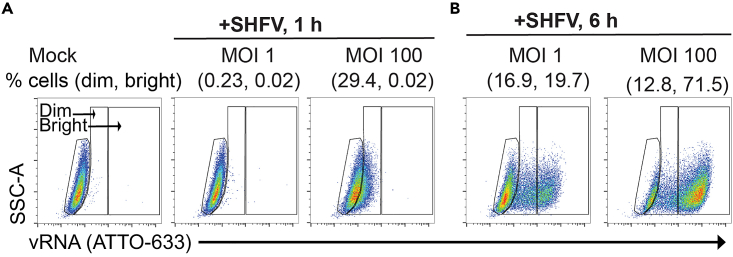
Example of results from cells infected with different MOIs over a time course (A and B) MA-104 cells were exposed to simian hemorrhagic fever virus (SHFV) at a multiplicity of infection (MOI) of 1 or 100. At time points of (A) 1 h and (B) 6 h post-exposure, the cells were harvested and analyzed with RNA fluorescence *in situ* hybridization flow cytometry (RNA FISH-Flow). Samples were hybridized with an SHFV ORF1a-specific ATTO-663 probe set. All flow-cytometric events were first gated on forward vs side scatter (FSC-A × SSC-A) properties, followed by singlet discrimination. All gates were drawn based on mock-infected cells, and the percentage of cells positive for viral RNA (vRNA) in the dim and bright gates were derived. This figure has been adapted from previous work.^1^

Next, we sought to validate that “dim gates” are indeed due to low levels of vRNA. We exposed cells to virus at different MOIs and harvested them over a time course. Following a 1-h incubation with a low MOI (MOI = 1), we did not observe any fluorescence signal; this is likely due to the threshold sensitivity of the assay. However, at a high MOI (MOI = 100), we observed a shift of the population into the dim gate (Figure 5A). When the cells had incubated for 6 h, the vRNA had robustly replicated and most events fell within the bright gate (Figure 5B). Based on this information, we surmise that this assay can discriminate between early events in virus infection (cell entry prior to RNA replication) and is most robust after vRNA replication occurs.

To visually assess viral infection in cells, many researchers turn to using viruses that have been genetically manipulated to encode a fluorescence reporter gene (e.g., enhanced green fluorescent protein [eGFP]). Achieving this first requires the generation of an infectious clone, i.e., a full-length DNA virus genome—usually encoded on a plasmid—that can be manipulated and from which infectious virus can be rescued following transfection of cells *in vitro*. Important considerations include whether the fluorescent reporter is stably maintained in the virus genome and its overall effects on the fitness (i.e., replicative capacity) of the virus. Given the substantial effort needed, and the potential caveats that come with generating fluorescent reporter viruses, we sought to determine whether RNA FISH-Flow could serve as an alternative method for the rapid measurement of viral infection in cells.

**Figure 6:**
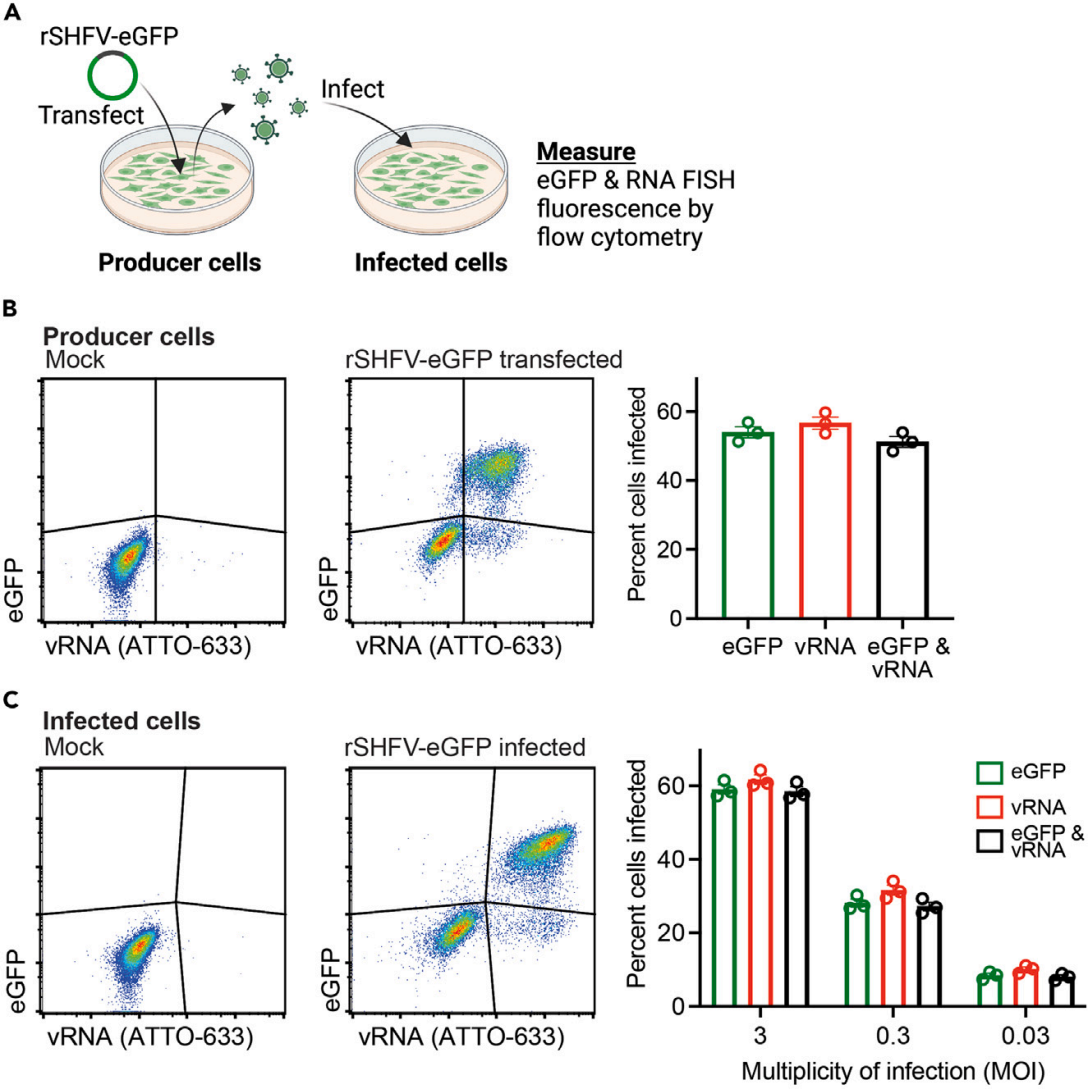
Comparison of RNA fluorescence *in situ* hybridization flow cytometry (RNA FISH-Flow) and standard flow cytometry using a recombinant infectious SHFV reporter virus clone (A) Schematic of the experimental setup to compare virus-encoded eGFP and RNA FISH-Flow measurements using cells transfected with a recombinant simian hemorrhagic fever virus expressing eGFP (rSHFV-eGFP)-encoding cDNA launch plasmid^6^ (producer cells) and cells infected with rSHFV-eGFP (infected cells). (B and C) (B) MA-104 cells were transfected with rSHFV-eGFP-encoding plasmid or (C) exposed to rSHFV-eGFP and then processed for RNA FISH-Flow 72 h after plasmid transfection or 24 h after virus exposure. (B and C) All flow-cytometric events were first gated on forward vs side scatter (FSC-A × SSC-A) properties, followed by singlet discrimination. All gates were drawn based on mock-treated cells, and the percentage of cells positive for viral RNA (vRNA) and enhanced green fluorescent protein (eGFP) were derived. The data show the mean ± SEM from three biological replicates.

To achieve this, we first transfected cells with a recombinant SHFV expressing eGFP (rSHFV-eGFP)-encoding cDNA-launch plasmid.^6^ Then we measured the percent of vRNA-expressing cells by RNA FISH-Flow and compared that to the percent of eGFP-positive cells enumerated by flow cytometry (Figure 6A). In rSHFV-eGFP transfected “producer” cells, there were no differences between the percentages of eGFP or vRNA-positive cells (Figure 6B). Next, using an rSHFV-eGFP infectious virus stock, we exposed cells to virus at various MOIs and again measured the percentage of eGFP/vRNA-positive cells by flow cytometry (Figure 6A). Consistent with the data in virus producer cells, RNA FISH-Flow was equivalent to eGFP in discriminating infected versus uninfected cells (Figure 6C). From these data, we conclude that RNA FISH-Flow is a suitable alternative to fluorescent reporter viruses in rapidly measuring infection kinetics by flow cytometry.

Multicolor flow cytometry has revolutionized many areas of research by enabling the detection of multiple protein targets at the individual cell level. We have successfully performed RNA FISH-Flow alongside surface antibody staining, further enabling simultaneous RNA and protein profiling by flow cytometry. While additional optimizations are still needed, we suspect that RNA FISH-Flow, when coupled with antibody staining, will be a powerful experimental tool for many researchers.

## Limitations

This procedure outlines an RNA fluorescence *in situ* hybridization-based approach to enumerate virus-infected cells. This approach is limited in that it may not be directly applicable to studying infection by viruses with double-stranded DNA or RNA genomes. Double-stranded genomes must first be denatured to enable probe access, and this will likely require hybridization temperatures >37°C. This protocol may have limited ability to detect short genome segments (<1 kb) of vRNA that cannot accommodate 48 RNA FISH-Flow probes. Using fewer than 48 probes may reduce the overall sensitivity of the assay. Additionally, this technique is most robust when detecting replicated vRNA. Detection of viral genomes produced during virus latency, for instance, may be inefficient due to limited quantities of vRNA present.

## Troubleshooting

### Problem 1

Low numbers of cells are detected during flow-cytometry acquisition, despite staining the recommended 1 × 10^5^ to 1 × 10^6^ cells.

### Potential solution

To improve cell recovery, the number of cells may be increased. However, we have found that cells are most often lost post-hybridization because cell pellets are easily dislodged during washes. We suggest tilting the plate to draw liquid out from the sides of the wells. It is not necessary to pull off 100% of the liquid if this would disturb the cells. We have also found that using a swinging bucket centrifuge rotor with a longer radius (i.e., >15 cm) generates a more compact pellet that is centered in the middle of a v-bottom plate well; this helps reduce cell loss during washing steps.

### Problem 2

Low numbers of RNA-positive cells are detected or fluorescent signal is weak.

### Potential solution

**Figure 7:**
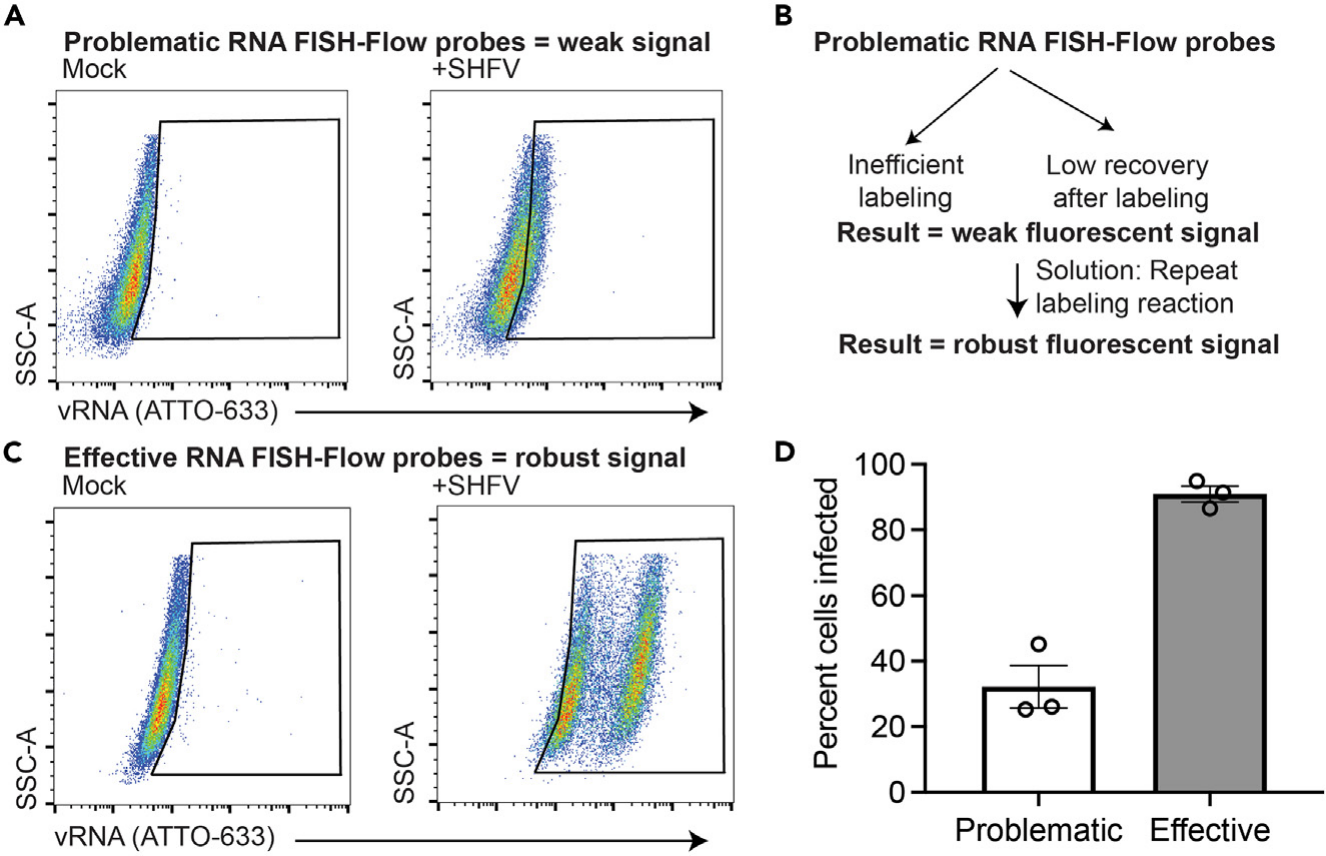
Example of results demonstrating weak signal and possible solutions (A) MA-104 cells were exposed to wild-type SHFV at a multiplicity of infection (MOI) of 3. Cells were harvested 24 h after exposure and processed for RNA FISH-Flow. Flow cytometric analysis of virus-infected cells revealed a weak shift in fluorescence. (B) This weak signal may be due to inefficient labeling or poor RNA FISH-Flow probe recovery after labeling. Ultimately, we repeated the labeling reaction. (C) The infection experiment was repeated as described in “A.” This newly labeled RNA FISH-Flow probe set exhibited robust viral RNA (vRNA) detection as indicated by a robust shift in fluorescence intensity. (A and C) All flow-cytometric events were first gated on forward vs side scatter (FSC-A × SSC-A) properties, followed by singlet discrimination. (D) All gates were drawn based on mock-exposed cells, and (D) the percentage of cells positive for viral RNA (vRNA) were derived. The data show the mean ± SEM from three biological replicates.
• Repeat with modifications to centrifugation times. If the recovery efficiency is below 50%, then the following modifications can be performed:
  ○ Increase the centrifugation time from 15 min at 4°C to 1 h at 4°C to improve recovery of RNA FISH-Flow probes.
  ○ Take care during washes to not disturb the pelleted RNA FISH-Flow probes. The ethanol washes should be added slowly to the side of the tube opposite of the pellet. The microfuge tube should always be maintained in the same orientation in the centrifuge so that the pelleted RNA FISH-Flow probes concentrate in the same location of the tube.
  ○ If you notice that some of the pellet is being dislodged during these washes, increase the centrifugation times from 1 min at 4°C to 15 min at 4°C to fully re-pellet the RNA FISH-Flow probes.
• Troubleshoot the reaction conditions. If the fluorescence ratio values of the labeled RNA FISH-Flow probes are not clearly distinct from the unlabeled oligonucleotides, then the following modifications can be performed:
  ○ It is possible that the TdT enzyme is compromised. Consider ordering a new enzyme and repeating the labeling reaction.
  ○ Ensure that the fluorescent dye-labeled ddUTPs were added to the reaction mixture. When added, the probe labeling reaction will appear colored.
  ○ Ensure that all reagents were added to the labeling reaction (e.g., buffer, enzyme).

• Optimize infection conditions. We recommend that the procedure first be optimized by assaying cells at different time points to identify a time coinciding with peak vRNA replication. This can be done by adjusting the MOI and/or time of cell collection and analysis. If it is determined that the experimental conditions are not a factor, the problem may be due to issues that arose during the RNA FISH-Flow probe labeling and purification steps (see below).
• Repeat the probe labeling procedure. On occasion, an RNA FISH-Flow probe set is generated that has a weak fluorescent signal, potentially resulting in underestimation of the percentage of vRNA-positive cells (see example in Figure 7A). A weak fluorescent signal is potentially due to two factors: (1) inefficient labeling or (2) low recovery of the RNA FISH-Flow probes after labeling (Figure 7B). We recommend following the procedures outlined in the section titled “quality control: spectroscopic analysis of RNA FISH-flow probes” to distinguish between these two different factors.

In general, we find that simply repeating the probe labeling procedure—paying close attention to the above factors—results in an effective RNA FISH-Flow probe set (as demonstrated in Figures 7C and 7D).

• Extend RNA FISH-Flow probe hybridization times and/or probe quantity. If the above conditions do not improve the signal, consider the following modifications:
  ○ If the RNA FISH-Flow probe was first diluted 1:10 prior to its addition to the hybridization buffer (0.05 μL delivered per well), instead use an undiluted volume of probe (0.5 μL delivered per well; see section on probe hybridization, step 43).
  ○ If a 1 h hybridization time was used, lengthen this time to 16 h (see section on probe hybridization, step 44). This increase may result in more complete hybridization of the RNA FISH-Flow probe set to its target.
• Re-design the RNA FISH-Flow probes. If the above solutions still do not improve the fluorescent signal, it is possible that the RNA FISH-Flow probes did not hybridize to the target. Double-check whether the RNA FISH-Flow probes were designed anti-sense to your target sequence. Also, consider re-designing the probes to target a different location in the viral genome.

### Problem 3

Non-specific signal is masking the specific detection of viral RNA.

### Potential solution

If the designed RNA FISH-Flow probes have complementarity to mRNAs expressed in the cells being assayed, it’s possible that a non-specific signal generated from off-target probe hybridization may mask the specific detection of vRNA. In a previous report, we described a similar technique—single molecule RNA FISH (smFISH)—that enables visual detection of an RNA FISH signal by microscopy.^1^ Briefly, cells fixed to coverslips are hybridized with RNA FISH-Flow probes, washed, and then imaged by confocal microscopy. In using this technique, it can be visually determined whether non-specific background signal is being generated following probe hybridization to uninfected cells. If this is indeed the case, either (1) extend the number of washing steps following hybridization to remove unbound probe or (2) if off target RNA FISH-Flow probe hybridization is of concern, redesign the probes to target a different region of the virus genome.

## Resource availability

### Lead contact

Further information and requests for resources and reagents should be directed to, and will be fulfilled by, the lead contact, Sara Sawyer (ssawyer@colorado.edu).

### Materials availability

This study did not generate new unique reagents.

### Data and code availability

This paper does not report original code.
